# Clinical Applicability of Electrical Impedance Tomography in Patient-Tailored Ventilation: A Narrative Review

**DOI:** 10.3390/tomography9050150

**Published:** 2023-10-18

**Authors:** Serge J. H. Heines, Tobias H. Becher, Iwan C. C. van der Horst, Dennis C. J. J. Bergmans

**Affiliations:** 1 Department of Intensive Care Medicine, Maastricht University Medical Centre+, 6229 HX Maastricht, The Netherlands; iwan.vander.horst@mumc.nl (I.C.C.v.d.H.); d.bergmans@mumc.nl (D.C.J.J.B.); 2 Department of Anesthesiology and Intensive Care Medicine, Campus Kiel, University Medical Centre Schleswig-Holstein, 24118 Kiel, Germany; tobias.becher@uksh.de; 3 Cardiovascular Research Institute Maastricht (CARIM), Maastricht University, 6229 HX Maastricht, The Netherlands; 4 School of Nutrition and Translational Research in Metabolism (NUTRIM), Maastricht University, 6229 ER Maastricht, The Netherlands

**Keywords:** acute respiratory distress syndrome, COVID-19, electrical impedance tomography, end-expiratory lung volume, ventilation inhomogeneity, ventilatory monitoring, personalized ventilation, positive end-expiratory pressure, prone positioning

## Abstract

Electrical Impedance Tomography (EIT) is a non-invasive bedside imaging technique that provides real-time lung ventilation information on critically ill patients. EIT can potentially become a valuable tool for optimising mechanical ventilation, especially in patients with acute respiratory distress syndrome (ARDS). In addition, EIT has been shown to improve the understanding of ventilation distribution and lung aeration, which can help tailor ventilatory strategies according to patient needs. Evidence from critically ill patients shows that EIT can reduce the duration of mechanical ventilation and prevent lung injury due to overdistension or collapse. EIT can also identify the presence of lung collapse or recruitment during a recruitment manoeuvre, which may guide further therapy. Despite its potential benefits, EIT has not yet been widely used in clinical practice. This may, in part, be due to the challenges associated with its implementation, including the need for specialised equipment and trained personnel and further validation of its usefulness in clinical settings. Nevertheless, ongoing research focuses on improving mechanical ventilation and clinical outcomes in critically ill patients.

## 1. Introduction

Mechanical ventilation is used in intensive care units to support patients with respiratory failure. However, this intervention has potential adverse effects, referred to as ventilator-associated lung injury (VALI). Over the past two decades, extensive research has focused on lung-protective ventilation for mitigating VALI. ARDSNet investigators published influential work in 2000 highlighting the importance of avoiding high tidal volumes in patients with acute respiratory distress syndrome (ARDS) [1]. Subsequent studies investigating high vs. low levels of positive end-expiratory pressure (PEEP) have consistently failed to demonstrate a reduction in mortality or have been refuted by other studies [2,3,4]. Various ventilation strategies, such as stress index [5,6], transpulmonary pressure [7,8], and pressure–volume curves, have been explored to determine the optimal PEEP setting [9,10]. Other frequently used bedside parameters to evaluate the effect of recruitment manoeuvres and PEEP settings are variables that reflect changes in respiratory system compliance or oxygenation [11,12,13,14]. However, these parameters are all based on global pulmonary function and do not provide regional information. Therefore, an individualised patient-centred approach for the adjustment of tidal volume and PEEP setting seems favourable. Depending on the heterogeneous distribution of the fluid-filled and atelectatic alveoli, different lung regions are prone to collapse or overdistension. Even with low driving pressure, a low tidal volume can generate local high lung strain [15].

Electrical impedance tomography (EIT) is a non-invasive, radiation-free, bedside monitoring tool that provides functional images of the lung with a relatively low spatial but very high temporal resolution. It was invented nearly 40 years ago [16] and uses small alternating currents to generate images that represent the regional distribution of resistivity within a body. The term tomography refers to imaging the volume within the body by penetrating energy from the outside. Impedance is a complex quantity that specifically applies to alternating current circuits. It includes both a real part (resistance) and an imaginary part (reactance). In clinically available chest EIT systems, 16–32 electrodes are attached around the patient’s chest circumferences, small alternating currents are applied between pairs of electrodes, and the resulting voltage differences are recorded by the other electrodes. The internal conductivity distribution within the chest was estimated using the measured voltages in an iterative process to determine the internal conductivity distribution that best matches the measured voltages. According to the incorporated image reconstruction algorithm, this process results in a two-dimensional tomogram. One EIT frame generates one reconstructed image, usually with a 32 × 32-pixel matrix, at a given time point during breathing. EIT allows clinicians to monitor the lung response to any ventilator setting adjustment on a breath-by-breath basis, enabling visualisation of regional alveolar overdistension and collapse, ventilation delay, and flow [17,18,19]. By determining the regional ventilation distribution, EIT can individually optimise the ventilator settings and probably improve patient outcomes. This review focuses on the clinical applicability, indices, diagnostic applications, and limitations of lung EIT in adults, including its use during the COVID-19 pandemic. EIT’s technical operating principles of the EIT are described in detail [20,21,22,23]. This review focuses on lung ventilation solely, not on perfusion.

## 2. Clinical Applications

### 2.1. Positive End-Expiratory Pressure and Tidal Volume Settings

#### 2.1.1. Calculation of Alveolar Overdistension and Collapse

The optimal PEEP for individual patients at a particular time within the treatment period remains disputed [24,25]. Assessment of proxy parameters such as oxygenation, best compliance, stress index, and low-flow pressure–volume curves may be misleading, as they are all based on global measures that exclude regional overdistension, collapse, or atelectrauma, especially in patients with ARDS because it is a heterogeneous process. Different regions of the lung have varying degrees of disease. According to Gattinoni et al., optimal PEEP is defined as the best compromise between regional overdistension and collapse. On the other hand, he stated that one “optimal” PEEP for the whole lung does not exist [26]. Regional overdistension and alveolar collapse can be visualised using EIT by calculating the regional compliance [17]. During mechanical ventilation, compliance can be calculated by dividing tidal volume by driving pressure. Electrical impedance tomography can monitor local impedance changes; in this way, local volume changes can be estimated by regional changes in lung impedance. Regional compliance can then be calculated by dividing regional tidal impedance variation by driving pressure [27,28]. In clinical practice, the most widely used method for titrating PEEP and tidal volume with EIT is the regional compliance-based approach [17]. The practical approach includes performing a decremental PEEP trial starting from the highest clinically acceptable PEEP level and then reducing the PEEP in small steps (e.g., 2 cmH_2_O) until the lowest clinically acceptable PEEP level is reached [29]. With this approach, the EIT can assess derecruitment and end-inspiratory overinflation within the two-dimensional electrode plane during a decremental PEEP trial.

**Figure 1:**
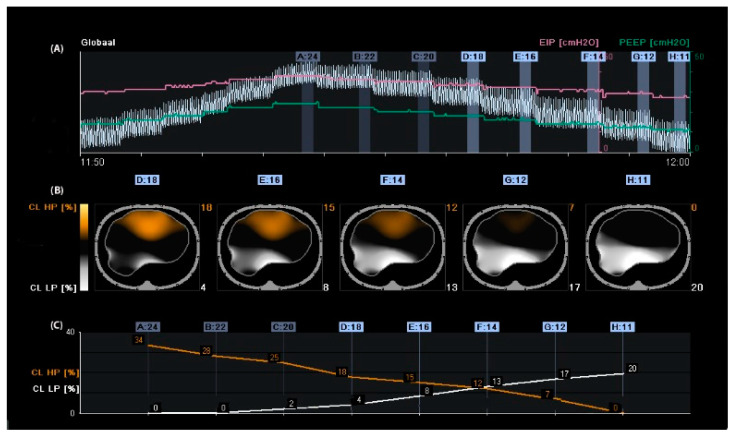
Regional compliance changes during the decremental positive end-expiratory pressure (PEEP) trial. Panel (**A**): Time course of the global impedance signal during an incremental and decremental PEEP trial. The decremental PEEP trial started from a PEEP of 24 cmH_2_O until a PEEP of 11 cmH_2_O. The last breaths of each PEEP step were averaged and used to analyse regional compliance. The green line represents the PEEP level, and the pink line represents the end-inspiratory pressure. Panel (**B**): Visualisation of relative compliance loss toward higher PEEP levels (CL HP, orange), which could be interpreted as relative alveolar overdistension, and compliance loss towards lower PEEP levels (CL LP, white), which could be interpreted as relative alveolar collapse. Panel (**C**): Time course of CL HP (orange) and CL LP (white). The PEEP level closest to the intersection of both lines represents the “best compromise” between alveolar overdistension (12%), collapse (13%), and PEEP of 14 cmH_2_O. The PEEP level with a CL LP of ≤5% would result in a PEEP of 18 cmH_2_O, resulting in a CL HP of 18%; in this case, the tidal volume would be reduced.

Titrating PEEP based on regional compliance measurements is difficult because both overdistension and collapse can result in decreased compliance but would require opposite titration strategies. However, optimal regional compliance at different PEEP settings differs between the cranial and caudal levels for dependent and non-dependent lung regions [30]. Nevertheless, this method has been successfully applied in several studies [31,32,33,34,35,36,37]. Based on this method, PEEP is frequently set at the “crossover point”, representing the “best compromise” between alveolar overdistension and alveolar collapse [38,39,40,41]. Hsu et al. showed that selecting PEEP according to this method resulted in a lower PEEP, lower driving pressure, and higher survival rate compared with PEEP set at the pressure where maximal hysteresis was reached during a low-flow pressure–volume loop in moderate-to-severe ARDS [42]. However, the best balance between alveolar overdistension and collapse could result in a large amount of overdistension in combination with a large amount of alveolar collapse (i.e., overdistension and collapse coexist), particularly in patients with ARDS because of the heterogeneity of the diseased lung [33]. Therefore, others have defined optimal EIT-guided PEEP as alveolar collapse ≤ 5% [29,30] (Figure 1). Simultaneously, if a large amount of overdistension exists, the tidal volume (driving pressure) can decrease.

The debate regarding optimal PEEP remains unresolved, and using proxy parameters such as oxygenation and compliance may be misleading due to the heterogeneous nature of ARDS. The most reliable method for titrating PEEP is the regional compliance-based approach using EIT, which can visualise and calculate regional overdistension and alveolar collapse.

#### 2.1.2. Positive End-Expiratory Pressure Based on Changes in End-Expiratory Lung Impedance

**Figure 2:**
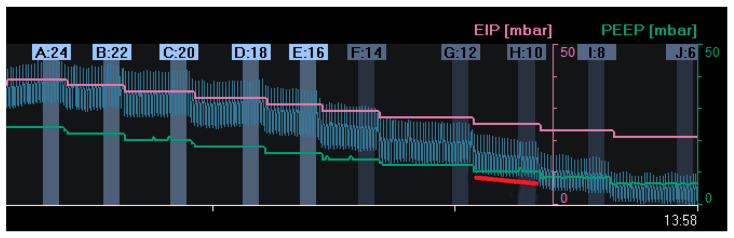
Changes in end-expiratory lung impedance (EELI): A decrease in EELI at different decremental PEEP levels reflects a stepwise decrease in end-expiratory lung volume with lower PEEP. At a PEEP level of 10 mbar (H:10), there was a gradual decrease in EELI (red line), reflecting alveolar derecruitment. (EIP = End Inspiratory Pressure; PEEP = Positive End Expiratory Pressure).

Another way to titrate PEEP and quantify lung recruitment is by measuring changes in end-expiratory lung impedance (EELI) [43]. Changes in tidal impedance have been correlated with regional tidal volumes [44,45]. Therefore, changes in EELI may be used to quantify regional changes in end-expiratory lung volume [46,47]. However, when EIT is measured at only one thoracic level, there is only moderate agreement between changes in EELI and end-expiratory lung volume during a PEEP trial [48]. As shown in Figure 1, an increase in PEEP resulted in higher EELI (end-expiratory lung volume). During a decremental PEEP trial, a gradual decrease in EELI at a fixed PEEP level may indicate loss of end-expiratory lung volume, which can be interpreted as derecruitment (Figure 2). In this case, PEEP can be set to a higher level where a decrease in EELI does not occur [31,49,50]. However, this method is less accurate; changes in the EELI at the bedside are global or divided into a few regions of interest and not pixel-wise. Furthermore, changes in EELI are sensitive to artefacts. Currently, alternating-pressure mattresses are commonly used in intensive care to prevent pressure ulcers. These can cause substantial changes in EELI, which cannot be explained by changes in end-expiratory lung volume [51].

A simple way to counteract this problem is to set the mattress to static mode before starting the EIT measurement. Unfortunately, these artefacts also occur during patient movement [52], which may be more difficult or impossible to address in clinical practice. Intravenous fluid admission can cause substantial changes in EELI [53,54]. Positive end-expiratory pressure titration based on the EELI trend suggests higher PEEP settings than the regional compliance method [55], which may be because, in contrast to the latter, it only assesses lung recruitment and derecruitment but provides no information about regional lung overdistension. Furthermore, it was impossible to compare changes in EELI using repeated measures at different time points. Skin conditions, electrode position, and environmental conditions influence the baseline frames and corresponding EELI values. Therefore, EELI values are not comparable at different time points when the electrode belt is detached and reattached to a patient [56].

However, measuring end-expiratory lung impedance changes (EELI) can provide information on lung recruitment but may not be accurate for detecting lung overdistension. It is also challenging to interpret and compare changes over time due to the global or regional nature of the changes and the presence of artefacts.

#### 2.1.3. Setting PEEP in Patients with Spontaneous Breathing Activity

In mechanically ventilated patients, early restoration of spontaneous breathing is beneficial for improving oxygen delivery and shortening the duration of mechanical ventilation. Excessive spontaneous breathing efforts, however, may cause additional harm via a variety of mechanisms that are nowadays referred to as “patient self-inflicted lung injury” (P-SILI) [57]. The pendelluft phenomenon, which is the movement of air within the lung from non-dependent to dependent regions without change in tidal volume, can be visualised by EIT. This phenomenon can cause cyclic alveolar recruitment and can result in local trauma, mainly in the dependent lung fields [58]. Applying higher PEEP levels may decrease the magnitude of spontaneous effort and improve lung ventilation homogeneity by opening up partially closed alveoli, suggesting that this may lead to less injurious ventilation [59,60]. In contrast, healthy lung tissue may be overdistended when PEEP levels are too high, inducing ventilator-associated lung injury [61]. Thus, determining the optimal PEEP level is challenging for patients undergoing assisted mechanical ventilation.

The drawback of most currently available algorithms for titrating PEEP is that their use is limited to patients on controlled mechanical ventilation. The regional compliance-based approach for calculating the level of alveolar overdistension and collapse is not readily feasible during assisted mechanical ventilation or spontaneous breathing. A reliable assessment of respiratory system compliance is required to obtain valid results. Elimination of spontaneous breathing requires neuromuscular paralysis or deep sedation. A fundamental assumption of the EIT respiratory system compliance measurement is that pressure changes are uniform throughout the lung when the flow reaches zero after inspiration and expiration. Within spontaneous breathing, the negative inspiratory pleural pressure swing following diaphragmatic contraction is not evenly distributed across the lungs because it acts mainly on the dorsal (dependent) lung regions, which could result in inaccurate quantification of the regional respiratory system compliance calculation during spontaneous breathing [62].

**Figure 3:**
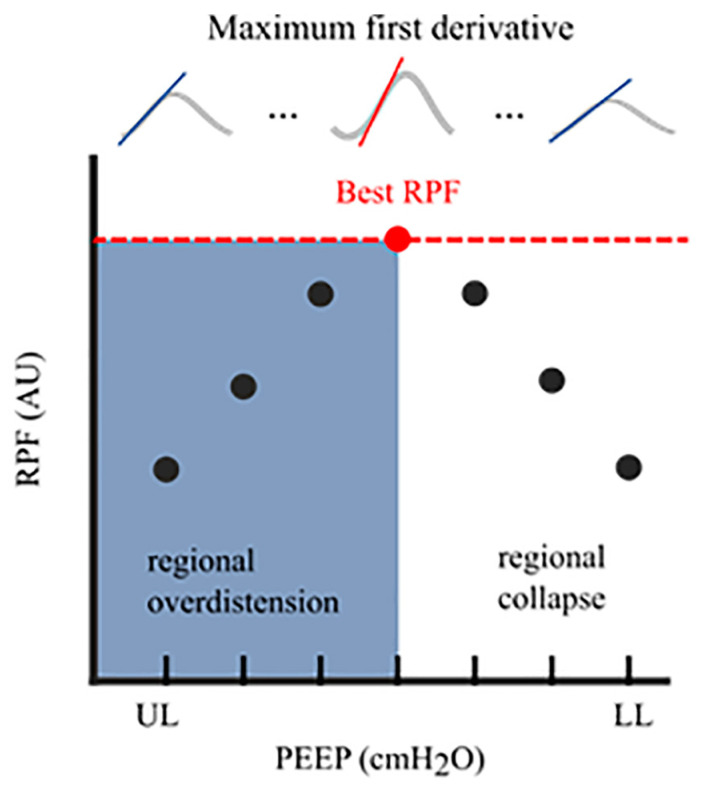
Regional Peak Flow (RPF): In each aerated pixel, the maximum first derivative (i.e., regional peak flow) was calculated for all segmented breaths at an arbitrary PEEP level. This resulted in median regional peak flow per PEEP level. The highest peak flow reflected the optimal PEEP level with the lowest level of alveolar collapse and alveolar overdistension. The cumulative collapse and overdistension rates were calculated identically to Costa’s algorithm [17]. RPF, regional peak flow; AU, arbitrary unit; UL, upper limit; LL, lower limit; PEEP, positive end-expiratory pressure.

A promising new algorithm was recently developed for quantifying regional lung mechanics, independent of a stable plateau pressure phase, based on the regional peak flow using EIT. The highest regional peak flow was calculated during a decremental PEEP trial (similar to the regional compliance-based approach), and regional alveolar overdistension and collapse were calculated in patients undergoing assisted mechanical ventilation or spontaneous breathing efforts (Figure 3). This method was validated in a prospective cohort of mechanically ventilated patients with COVID-19 ARDS in a controlled mechanical ventilation mode. There was a good correlation between the levels of alveolar overdistension and collapse based on the highest regional peak flow and alveolar overdistension and collapse based on the regional compliance-based approach [19].

Although end-expiratory lung impedance can be used to titrate PEEP and quantify lung recruitment, it is less accurate and sensitive to artefacts. Furthermore, most PEEP titration algorithms are limited to mechanically ventilated patients. However, a new algorithm based on regional peak flow using EIT shows promise for quantifying regional lung mechanics in patients undergoing assisted mechanical ventilation or spontaneous breathing.

## 3. Measures of Ventilation Distribution

### 3.1. Anterior-to-Posterior Ventilation Ratio (Impedance Ratio)

A standard and easy-to-use measure of anterior–posterior ventilation distribution is the anterior-to-posterior ventilation ratio (A/P ratio) [63,64]. This ratio was initially called the Impedance Ratio (IR) [65], which is still used in the literature sometimes. The tidal impedance difference (the impedance change between the end and the beginning of inspiration) in the non-dependent part of the lung was divided by the tidal impedance difference in the dependent part of the lung. A decrease in the A/P ratio indicates an increase in ventilation in the dependent part of the lung above the non-dependent part and a ventilation distribution shift from the ventral to the dorsal parts of the lung. An A/P ratio of “1” indicates equal ventilation distribution in the ventral part of the lung compared to that in the dorsal part. As the ventral and dorsal halves of the lung are not identical, the A/P ratio is not expected to be equal to one, even in the case of healthy lungs. In ARDS patients, a higher PEEP results in a decrease in the A/P ratio [66].

In addition to a higher PEEP level, enhanced spontaneous breathing may increase the proportion of tidal ventilation reaching the dependent lung regions in patients with ARDS, likely indicating a higher efficiency of the posterior diaphragm that leads to a decrease in the A/P ratio [67]. In neonates, the A/P ratio has been used to select the “optimal PEEP” by choosing a PEEP level in which the A/P ratio is closest to “1” [63]. Another study on patients with early mild ARDS compared the optimal PEEP guided by the open lung concept strategy with the ARDS network protocol [1,68]. The anterior-to-posterior ventilation ratio was used to indicate the effect of the open-lung concept, and the PEEP selected for the open-lung concept was significantly higher than that of the ARDS network table. The A/P ratio decreased after applying the open-lung concept. The A/P ratio has been widely used; however, this measure is less robust than other measures, such as the centre of ventilation, and its specificity of ventilation shift is much smaller than that of the conventional centre of ventilation [23].

In short, the A/P ratio (A/P ratio) is a standard and easy-to-use measure of ventilation distribution in the lungs. An A/P ratio of “1” indicates equal ventilation distribution between the ventral and dorsal parts of the lung. Higher PEEP decreased the A/P ratio in patients with ARDS, indicating more homogeneous ventilation. However, the A/P ratio was less robust than other measures, such as the centre of ventilation.

### 3.2. Centre of Ventilation

**Figure 4:**
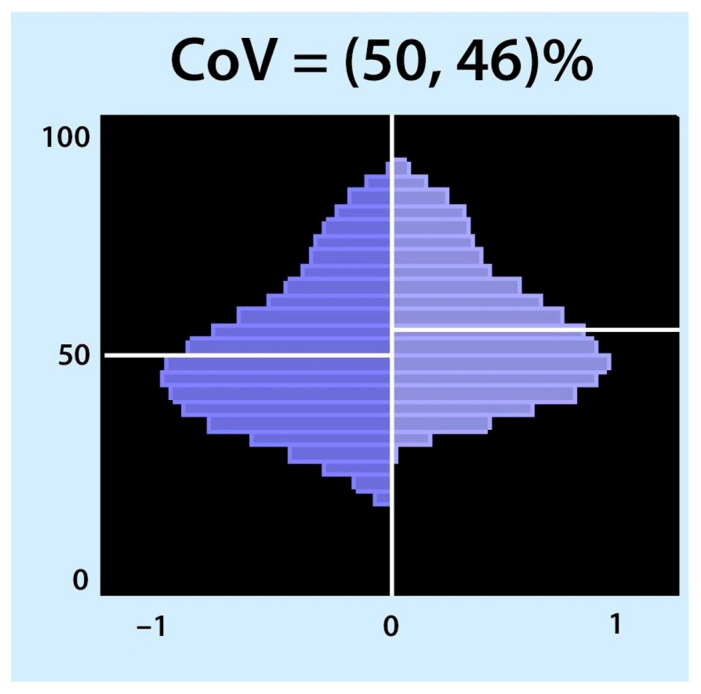
Centre of ventilation (CoV): The CoV examines the right and left halves of the ventilated area separately. Each half was divided into equally spaced horizontal regions of interest (ROI). The sum of the ventilation-related impedance changes for each ROI was calculated and presented as a bar. The results are displayed as two histograms: the right histogram represents the left lung (and vice versa). The location of CoV is indicated by a white horizontal line that divides the ventral and dorsal lung regions with equal impedance changes. Two percentages separated by a comma were specified. These percentages represent the dorsal-to-ventral ventilation distributions. The first percentage represents the left histogram (right lung), and the second is the right histogram (left lung). A percentage higher than 50 represents a shift in the ventilation distribution towards the dorsal regions.

The centre of gravity was used synonymously with the centre of ventilation (CoV) introduced by Frerichs et al. [69]. The term centre of gravity is not recommended because it is used in mechanics and is defined as the average location of the weight of an object [23]. CoV describes the distribution of ventilation between the ventral and dorsal lung regions. This calculation can be performed separately for the right and left lungs. In this case, the weighted mean was also calculated (considering the possible differences in the ventilation magnitudes for the left and right sides). A value of 50% represents equally distributed ventilation between ventral and dorsal regions. Higher values represent a shift of ventilation distribution towards the dorsal regions (Figure 4, bottom of the image), and lower values represent a shift towards the ventral regions. This may be confusing because in some papers, the values are inverted, meaning that 100% is the most ventral region [70]. CoV was first introduced to describe the ventilation distribution during spontaneous breathing before and after surgery and its difference compared with mechanically ventilated patients [69]. It is a sensitive index that describes alveolar recruitment during a decremental PEEP trial. Luepschen et al. showed that during a decremental PEEP trial, although respiratory system compliance was still increasing, CoV shifted to the ventral lung regions, coinciding with a decrease in PaO_2_ [71]. CoVs have been used as a measure of ventilation distribution in both experimental and clinical studies [72,73,74,75,76,77,78,79].

In short, the anterior-to-posterior ventilation ratio (A/P ratio) and centre of ventilation (CoV) are measures used to describe lung ventilation distribution, with CoV being more sensitive to alveolar recruitment and derecruitment during PEEP trials.

### 3.3. The Global Inhomogeneity Index

The most frequently used measure of ventilation inhomogeneity is the global inhomogeneity index (GI). It provides information on the overall degree of ventilation inhomogeneity without information on how it is distributed in the lungs [80]. Global inhomogeneity was calculated as the sum of the absolute differences between the median value of tidal variation and every pixel value divided by the sum of all impedance values for normalisation. Higher values denote a greater degree of inhomogeneity in the ventilation distribution. Extrapulmonary regions should not be included in the calculation; however, if only the ventilated area is included, areas belonging to the lung that are overdistended or not ventilated due to atelectasis will be missed, leading to erroneous results [81]. Therefore, it is crucial to define the lung area within a tidal image [82,83]. The GI is reliable for inter-individual comparisons [80]. GI has been used for PEEP titration in patients with healthy lungs, and no significant differences were found between the GI method and the best dynamic respiratory system compliance method or between GI and the compliance volume curve method (stress index). The optimal PEEP level for each patient was determined according to the lowest GI index, corresponding to the most homogeneous lung ventilation distribution. GI is superior to dynamic lung mechanics in spontaneously breathing patients, where reliable lung mechanics are difficult to obtain [82,84].

The GI can also be used during spontaneous breathing trials. Patients with diaphragmatic dysfunction have larger increments in the inhomogeneities of lung aeration than those without diaphragmatic dysfunction [85]. GI is highly correlated with lung recruitment; the percentage of recruitable lung regions decreases with a decrease in GI [86]. A high tidal volume may lead to a lower GI index, especially at a low PEEP, probably because of tidal recruitment [87]. After a recruitment manoeuvre in patients with ARDS, the GI did not change in non-responders to recruitment; however, in responders, the GI improved. Thus, GI can help identify responders to recruitment manoeuvres [50].

It should be noted that homogenisation of lung ventilation became synonymous with protective ventilation, assuming that reopened lung units can improve ventilation distribution by accommodating part of the tidal volume, thus minimising hyperinflation. However, in normal lungs with minimal collapse, the heterogeneity of lung ventilation is a physiological phenomenon mirrored by the heterogeneity of lung perfusion [88]. Therefore, the measures of ventilation inhomogeneity should be interpreted with caution. Solely trying to minimise inhomogeneity without limiting the upper level of PEEP may cause severe overdistension and is potentially harmful [89].

The global inhomogeneity index (GI) is a commonly used measure of ventilation inhomogeneity that reflects the degree of inhomogeneity in the overall ventilation distribution. However, caution should be exercised when interpreting these results, as homogenisation of lung ventilation should not be the only goal of protective ventilation.

## 4. Regional Ventilation Delay

**Figure 5:**
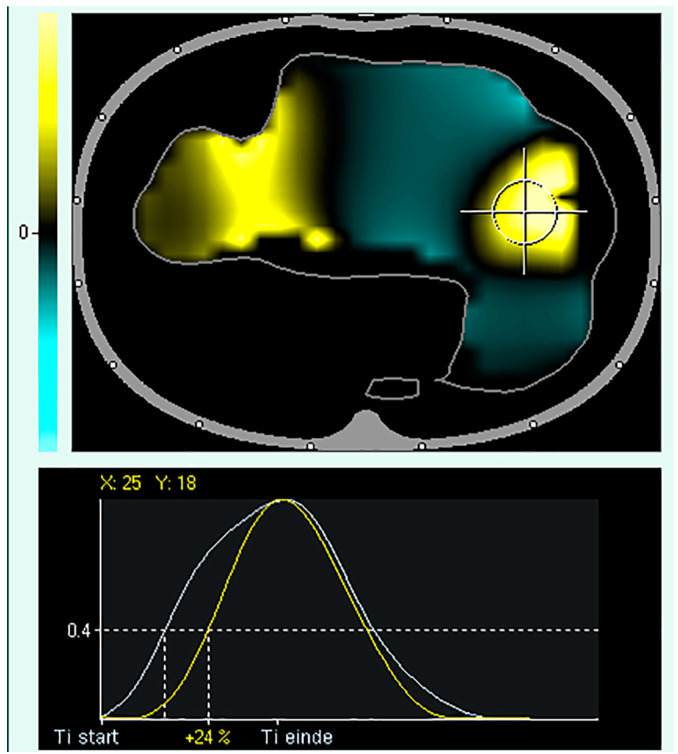
Regional Ventilation Delay (RVD). Upper panel: tidal image representing ventilation distribution (grey surrounding line of the ventilated area). The black regions indicate the beginning of inspiration simultaneously compared to the global beginning of inspiration; the yellow regions indicate a delayed and green region at the early beginning of regional inspiration compared to global inspiration. Lower panel: Delay in regional impedance change with a cutoff value of 40% set by the user. The white waveform represents the global start and end of inspiration, and the yellow waveform represents the start and end of inspiration of the pixel.

The regional ventilation delay (RVD) is the calculation of the delay between the global start of inspiration and the point in time at which the regional impedance curve reaches a certain impedance change threshold (Figure 5). Thus, RVD is used to identify lung regions with late opening, which could indicate the presence of cyclic opening and closing of the alveoli. Regional ventilation delay calculations have been applied during normal spontaneous breathing and conventional ventilation [90,91,92]. However, this calculation has not been validated for conventional control or support ventilation, where the inspiration time is short. Regional ventilation delay is determined during a low-flow manoeuvre, and the threshold for the inspiratory phase can be modified [18]. Global function tests such as a low-flow manoeuvre can only assess the overlapping information of several ventilatory units of different lung regions that differ in mechanical behaviour. Slow-flow inflation should promote sequential filling of different lung regions caused by alveolar recruitment of regions with different opening pressures. Regional ventilation delays during low-flow manoeuvres are useful in determining regional recruitment [18]. Regional ventilation delay inhomogeneity, therefore, provides a good estimate of the amount of tidal recruitment and may be useful for individualising ventilatory settings, such as PEEP [93,94,95,96]. The greatest drawback of performing a low-flow manoeuvre is that respiratory muscles must be inactive. Therefore, the patient needs to be deeply sedated and preferably paralysed [97,98]. To increase clinical applicability, reducing the volume delivered during a low-flow manoeuvre has been proposed [99]. More recently, there has been a trend of using lower doses of sedatives in intensive care patients to prevent muscle weakness, depression, and post-intensive care syndrome, all of which markedly affect patients’ quality of life after they leave the unit [100], making the usability of a slow-flow manoeuvre less applicable.

In short, the regional ventilation delay (RVD) measures the delay between the global start of inspiration and the regional impedance curve reaching a certain impedance change threshold, identifying regions with cyclic opening and closing. However, deep sedation and paralysis are required for low-flow manoeuvres.

## 5. Posture

Postural therapy has been widely accepted in critically ill patients to support ventilation redistribution towards the dependent lung areas, thus facilitating recruitment [101]. Electrical impedance tomography has the potential to guide physicians in positioning their patients according to their disease and lung condition [102]. Regional information on aeration in specific pulmonary regions by EIT might allow the discrimination of patients who will benefit from postural therapy, such as prone positioning, from those who will not. In the latter case, it could lead to a decrease in the delay in the initiation of other therapies, such as extracorporeal membrane oxygenation. The effect of body positioning on intrapulmonary tidal volume distribution can be easily assessed using EIT. The timing of the termination of prone positioning and lowering the PEEP setting was determined to prevent dorsal derecruitment as seen by EIT [103]; however, Spaeth et al. demonstrated that a higher PEEP level is required to prevent alveolar collapse in the prone position compared to the supine position [104]. A prolonged prone position in patients with ARDS results in a more homogeneous ventilation distribution and better oxygenation, probably because of better dorsal ventilation [105]. Homogenisation of ventilation distribution is much less dependent on the PEEP level in the prone position than in the supine position [106]. It has also been demonstrated that alveolar recruitment manoeuvres are more effective in the prone position [107]. Some investigators have also examined the changes in EELI when changing from supine to prone positioning [108]. However, these findings should be interpreted cautiously because changes in body position influence changes in EELI and are unrelated to changes in end-expiratory lung volume [52,56].

In the supine position, the electrodes on the back are pressed onto the skin because of the body weight, causing the skin–electrode resistance to be lower. When the patient is turned, the resistance of the dorsal electrodes increases, whereas the ventral electrodes have better contact and, thus, a lower resistance. This leads to changes in EELI but not in lung volume. However, the ventilation distribution can be reliably compared before and after prone positioning using two different baselines, thereby ignoring changes in EELI, and allowing only changes in ventilation distribution to be visualised. Reifferscheid et al. showed that posture significantly affected the distribution of regional tidal volume compared with the supine to sitting and right-lateral positions. More importantly, they showed that the reproducibility of regional ventilation determined by EIT, even after eight days, was good. For reproducibility during different measurements, they recommended carefully choosing the EIT examination location on the chest (note the intercostal space of the belt on the parasternal line and document typical anatomical landmarks) [109]. It must be considered that one needs to take some time is required for the regional ventilation distribution to stabilise after changing the position. It has been described that a 15 min stabilisation period should be allowed following any change in position before acquiring data in healthy volunteers (in both the anterior–posterior and left–right directions) [110]. Patients with pulmonary pathology are likely to require longer stabilisation times. The distribution of tidal ventilation is highly variable and influenced by body and neck position [111,112,113]. The prone position is the most commonly used therapy for posture to improve oxygenation and has been extensively used in patients with severe acute respiratory syndrome coronavirus 2 (SARS-CoV-2) infection.

In short, postural therapy in critically ill patients can be guided by electrical impedance tomography (EIT) to improve ventilation redistribution and prone positioning has been shown to improve ventilation homogenisation and recruitment in patients with acute respiratory distress syndrome (ARDS).

## 6. Belt Position

It is evident that if the patient has wounds or needs dressings where the belt has to be placed, EIT measurement is impossible because the electrodes must be in contact with the patient’s skin. However, contact with all the electrodes is unnecessary, depending on the device used. Electrical impedance tomography measurements are sometimes possible without contact with any electrodes. However, the quality of the measurements is reduced. Correct belt placement is crucial. If the belt is placed too low, the diaphragm can enter the EIT measurement field, inadvertently changing the ratio between the impedance change (delta Z) and the tidal volume. Such a change in delta-Z/mL tidal volume will result in an erroneous determination of lung volume change (changes in EELI reflecting changes in end-expiratory lung volume) if unrecognised [48]. During breathing, especially if the belt is placed in a more cranial position, the electrodes can move up and down the skin, resulting in negative impedance changes during inhalation due to changes in the electrode position (artefact). Therefore, it is important to select the appropriate electrode plane size, body position, and region of interest for analysis [114]. When EIT is used to estimate global parameters such as tidal volume or changes in end-expiratory lung volume, the belt is best placed around the patient’s chest at the fourth or fifth intercostal space measured at the parasternal line, resulting in the highest correlation between volume changes and impedance changes [44].

In patients with a known high intra-abdominal pressure, the belt could already be placed in a more cranial position. It is advisable to begin EIT measurements with a lower PEEP. If the belt is placed too low, it is recognised from the beginning. If the EIT measurement started with a high PEEP level and a decremental PEEP trial was performed, the diaphragm could enter the measurement field if the belt was placed caudally, which, in this case, would only be noticed at the end of the measurement. The PEEP level with the best regional compliance was different for the dependent and non-dependent lung regions, as well as for the caudal and cranial lung regions. The importance of the measurement location must be realised during clinical PEEP titration. Measuring EIT at different thoracic levels provides additional information on ventilation distribution in a larger part of the lung [30,115]. Although tidal volume and delta-Z correlate well [116], the volume-to-impedance ratio is not constant when volume or airway pressure is altered [117,118]. Therefore, electrode belt placement in the fifth intercostal space is not always ideal [119]. The repeated EIT measurements over time showed good reproducibility. However, the location of the belt attached to the chest must be carefully selected and marked [109].

In short, proper placement of the EIT belt on the patient’s chest is crucial because it can affect the quality and accuracy of measurements and contact with all electrodes may not always be necessary.

## 7. Diagnostic Applications

### 7.1. Airway Clearance Techniques

Electrical impedance tomography was used to evaluate the effects of airway clearance techniques. Endotracheal suctioning, which promotes derecruitment, is the most commonly used method. However, EIT has shown that a closed suctioning system only partially prevents derecruitment [120,121]. The dorsal regions are most affected by disconnection and suctioning, with a marked decrease in regional compliance. Another airway clearance technique is high-frequency percussive ventilation superimposed on mechanical ventilation, which promotes alveolar recruitment, as demonstrated by EIT [122].

### 7.2. Tube Misplacement and One-Lung Ventilation

Endobronchial intubation is one of the most common complications of endotracheal intubation during anaesthesia [123]. EIT may be useful for early diagnosis of untoward endobronchial intubation [124]. The feasibility of EIT for confirming the correct placement of a double-lumen tube (DLT) was studied by Steinmann et al. [125]. They concluded that EIT could be used for non-invasive online recognition of the misplacement of left-sided DLTs in the contralateral main bronchus. However, as EIT did not allow the detection of wrongly positioned endobronchial cuffs, it could not replace fibre optic bronchoscopy in the routine control of the DLT position. Nevertheless, since EIT allows reliable online diagnosis of grossly malpositioned DLT immediately after intubation, it could be of considerable clinical relevance since the incidence of initially misplaced left-sided DLTs into the right main bronchus was 12.5% in their study and varied between 7% and 24% [126,127]. Thus, EIT could also be useful for monitoring one-lung ventilation during closed chest conditions, especially if prolonged, such as during one-lung ventilation in intensive care. Tube displacement after changing the patient’s position during standard ICU care using a DLT is common, and detection and correction of DLT displacement, in this case, are crucial [128].

### 7.3. Detection of Pleural Effusion and Monitoring of Lung Re-Aeration after Aspiration of Pleural Effusion

EIT can detect pleural effusion by analysing phase-inverted impedance changes in dorsal lung areas. These are caused by step changes in the conductivity between the non-conductive lung and the highly conductive pleural fluid [129]. Furthermore, EIT can be used to evaluate re-aeration and re-ventilation after aspiration of pleural effusions. Re-aeration occurred immediately and was heterogeneous. Relief of compression after pleural aspiration creates higher transpulmonary pressure, favouring an increase in ventilation. If the ventilation at the side of the aspiration did not improve, the effusion was probably not compressing the lung or was caused by dysfunction of the diaphragm after a long period of compression due to pleural effusion. Occasionally, aeration decreases, as described by Alves et al. [130]. They hypothesised that this was due to the small airway opening and closing caused by the decrease in surfactant due to the chronically collapsed lungs. Small airway closure can result in trapped air (intrinsic PEEP) during expiration, leading to hyperinflation that would cause vigorous re-aeration but not re-ventilation. However, the effect of pleural effusion evacuation on lung aeration in mechanically ventilated patients cannot be evaluated effectively using EIT alone. In addition to the effects of reaeration, the increase in EELI due to pleural effusion evacuation may also be caused by the loss of conductive electrolytes (i.e., pleural effusion) adjacent to the EIT belt [131].

### 7.4. Early Detection of Pneumothorax

Costa et al. were the first to demonstrate the possibility of detecting the development and location of pneumothorax in quasi-real time using EIT in an experimental pig model. Pneumothoraces as small as 20 mL were detected, with a sensitivity of 100% and specificity of 95%. However, pneumothorax must develop or enlarge during EIT monitoring [132]. A rapid increase in local EELI can be an early sign of pneumothorax before all clinical signs. This indicates non-ventilated air in that specific region, followed by decreased ventilation distribution [133,134]. Patients with pneumothorax have a higher difference in ventilation distribution in the affected ventral quadrant than in the whole lung or dorsal part of the lung. Furthermore, ventilation distribution is more inhomogeneous in patients with pneumothorax than without [135]. Thus, EIT can be a useful warning tool to detect pneumothorax during high-risk procedures such as bronchoscopic biopsies or endobronchial lung-volume reduction valve placement.

### 7.5. Quantification of Pulmonary Oedema

The EIT can determine extravascular lung water during lateral body rotation by calculating the tidal variation differences between the left and right lungs in an experimental model. The extravascular lung water from the dependent part of the lungs is redistributed when the body position is changed. Thus, EIT can quantify pulmonary oedema at the bedside and differentiate between healthy and injured lungs [136]. However, this method cannot be used in human subjects with ARDS. None of the EIT measures correlated with lung oedema measures determined by transpulmonary thermodilution [137].

### 7.6. Monitoring Chronic Lung Diseases

There is the potential to evaluate the degree of airflow limitation in patients with chronic obstructive lung diseases, not only globally but also at the regional level. Pulmonary function testing can be performed at the bedside using EIT to evaluate progress and therapeutic effects in patients with pulmonary diseases [138]. It has been demonstrated that it is possible to show the response of bronchodilator therapy to regional EIT-derived lung function measures [139,140]. Regional lung function was more homogenous in healthy subjects than in those with chronic lung disease. Moreover, in patients with asthma, the regional ventilation distribution improves after bronchodilator administration, as demonstrated by EIT [141]. In addition, in patients with cystic fibrosis, EIT has been shown to deliver global and regional information on airway obstruction [142]; these findings are reliable and comparable with those of high-resolution computed tomography [143].

Assessing regional respiratory time constants using EIT is a promising approach for monitoring airflow obstruction in mechanically ventilated patients with COPD and ARDS [144]. In the future, it may become a useful tool for adjusting the ventilator settings in patients with clinically relevant expiratory airflow obstruction.

In short, EIT can be used to evaluate the effect of airway clearance techniques, monitor tube misplacement and one-lung ventilation, evaluate re-aeration and re-ventilation after aspiration of pleural effusion, detect pneumothorax, quantify pulmonary oedema, and monitor airflow obstruction in acute and chronic lung diseases. These applications range from the early diagnosis of complications to monitoring therapy progress in patients with pulmonary diseases. An overview of the key studies on the clinical use of EIT is presented in Table 1.

## 8. Limitations

The constraints and challenges associated with electrode belts were extensively described. EIT is not recommended in patients with cardiac pacemakers and electrically active implants such as implantable cardioverter-defibrillators.

Electrical impedance tomography signals can be inverted (out-of-phase impedance changes) in patients with pleural effusions. These are characterised by a paradoxical decrease in impedance during inspiration, followed by an increase during expiration. This might lead to an inaccurate interpretation of the EIT findings. Therefore, pleural effusion may affect the quantitative evaluation of regional lung ventilation using the EIT. Delta-Z cannot be used to estimate tidal volume in extremely heterogeneous states of pulmonary diseases such as unilateral empyema [145]. However, out-of-phase impedance changes can trigger further examination of pleural effusion and, in that way, may have a high diagnostic value [129]. Most EIT studies on lung pathology involve ARDS or experimental acute lung injury in animals, and only a few studies have been published on EIT and excessive fluid in the pleural space. During pleural fluid aspiration, increased electrical resistivity has been observed in patients with pleural effusion of cardiac origin [146,147]. Hahn et al. described a local decrease in resistivity during the installation of Ringer solution into the pleural cavity on regional lung ventilation in an experimental study [148]. An airless state may indicate alveolar collapse but may also be caused by pleural effusion [149]. Furthermore, fast intravenous administration of a saline bolus causes a significant decrease in EELI, which is not correlated with changes in end-expiratory lung volume and may be misleading. This limits the ability of critically ill patients to perform measurements over a longer period of time. However, fast intravenous saline administration does not affect regional lung ventilation distribution [53,54].

Electrical impedance tomography is precise when looking at regional ventilation and not overall lung volume because it only measures slices of the lung where the electrode plane is situated. Impedance changes were measured using lens-shaped chest slices. Its thickness increases towards the central region of the body (up to a thickness of approximately 12 cm). The assumption of a linear relationship between the change in global tidal impedance and tidal volume cannot be used to calculate the EELV when the EIT is measured at only one level just above the diaphragm [48]. Erlandsson et al. described a good agreement between impedance variation and tidal volume [43]. The slope between delta volume and delta impedance was very similar in a heterogeneous group of patients with different causes of respiratory failure. Although the correlation of impedance changes with changes in volume may be good, it may not be exact [47]. When EIT estimates global parameters such as tidal volume, the electrode plane should be placed between the fourth and fifth intercostal spaces for the highest correlation between tidal volume and tidal impedance difference (TID) [44]. It is calculated from the impedance change between the end and the beginning of inspiration (i.e., the tidal variation). Electrical impedance tomography reflects changes in the impedance of the lungs but not the absolute values.

EIT technology offers bedside visualisation of lung ventilation and dynamic changes in regional ventilation distribution instead of the static image from computed tomography (CT) [18]. CT is a high-resolution imaging technique that allows the visualisation of anatomical structures. However, this is not possible with EIT owing to its low spatial resolution. As mentioned above, the EIT visualises impedance changes. When no impedance changes occur in a certain lung area, this may be due to various causes, such as extreme overdistension, pneumothorax, atelectasis, or one-sided ventilation. These causes cannot be easily differentiated when considering EIT-based ventilation distribution alone.

Thus, EIT is not recommended for patients with pacemakers or electrically active implants. EIT signals can be inaccurate in patients with pleural effusion, and EIT can only measure regional ventilation rather than the overall lung volume. EIT offers bedside visualisation of lung ventilation but has a lower spatial resolution than CT. The number of electrodes used for EIT is limited, which affects resolution.

## 9. Electrical Impedance Tomography during the COVID-19 Pandemic

Severe acute respiratory syndrome coronavirus 2 is a strain of coronavirus that causes COVID-19 and is responsible for the COVID-19 pandemic that struck the world from 2019 onwards [150]. Some patients develop severe hypoxic respiratory failure, requiring ICU admission for respiratory support and mechanical ventilation. Gattinoni et al. described two phenotypes of COVID-19 patients: Type L, characterised by low elastance (high lung compliance), low ventilation/perfusion ratio, and low alveolar recruitment and Type H, defined by high elastance (low lung compliance), high right-to-left shunt, high lung weight, and high recruitability [151]. However, vigilance for premature phenotyping has been advocated because the disease and pulmonary interaction might change the appearance of the phenotype over time [152]. Therefore, it is crucial to adapt ventilatory support on a case-by-case basis over time. Electrical impedance tomography can detect different characteristics of the regional ventilation profile; therefore, EIT can be a helpful bedside tool for understanding the aetiology of hypoxaemia [153]. In an expert opinion in the Netherlands in 2020, the recommendation was to select the right level of PEEP using EIT, among other methods [154].

Our research group performed a unique number of EIT measurements in 80 patients during the first pandemic wave (analysing 334 EIT measurements) at the population level, showing that EIT-guided PEEP (the regional compliance-based approach [17]), alveolar overdistension (OD), alveolar collapse (CL), and dynamic respiratory system compliance (Cdyn) changed over the course of mechanical ventilation during SARS-CoV-2 infection in a prospective observational study. The focus was on serial EIT measurements, and we reported the EIT-derived pulmonary parameters over the course of mechanical ventilation in these patients. Optimal EIT-guided PEEP was determined at a level of CL ≤ 5% during a decremental PEEP trial. We showed that using EIT-guided PEEP, OD, CL, and Cdyn changes during mechanical ventilation for SARS-CoV-2 infection, suggesting that the whole population develops decreased compliance after a while. These changes were more unfavourable in non-survivors than in survivors. They demonstrated that PEEP titration is important both individually and over time [32].

Others used PEEP titration at the lowest PEEP above the intercept of curves representing the relative OD and CL in COVID-19 patients. They found that EIT-guided PEEP corresponded better with a high PEEP-FiO_2_ ALVEOLI table [13]. However, EIT-guided PEEP was lower in many cases and higher than that in the PEEP-FiO_2_ table [41,155]. Therefore, they concluded that PEEP should be personalised. No correlation was found between EIT-guided PEEP and FiO_2_ [156]. This was also the conclusion of a study comparing optimal PEEP based on the PEEP-FiO_2_ ALVEOLI [13], EIT, and transpulmonary pressure-FiO_2_ tables [157]. They found a poor agreement for the optimal PEEP. The optimal PEEP guided by EIT was based on the best balance between the OD and CL. Electrical impedance tomography-guided PEEP results in lower plateau pressure, mechanical power, transpulmonary pressure, higher static respiratory compliance, and homogeneity of ventilation [158]. The regional compliance-based approach for the EIT-guided PEEP setting was also used in a patient on venovenous extracorporeal membrane oxygenation during the pandemic [159,160]. Patients with severe COVID-19-related ARDS had respiratory characteristics compared to those without non-COVID-19 ARDS. However, in COVID-19-related ARDS patients, a higher PEEP level was required and had lower levels of overdistension compared with non-COVID-19 ARDS.

Patients with ARDS have been shown to benefit from early prone positioning if hypoxemia is severe and refractory [161,162]. Before the COVID-19 pandemic, the prone position remained remarkably underused [163,164]. Prone positioning is often used in COVID-19 patients, even in those who do not fulfil the usual (local) indications [165]. Perier et al. compared optimal PEEP based on the interception of the OD and CL curves in the supine and prone positions in patients with COVID-19 ARDS and non-COVID-19 ARDS. They found that the optimal PEEP in COVID-19 patients was similar in the supine and prone positions. However, in the supine and prone positions, the optimal PEEP was higher in COVID-19 patients than in non-COVID patients with ARDS. Furthermore, the response to PEEP on the PaO_2_/FiO_2_ ratio was similar in COVID-19 patients with high versus low respiratory system compliance [166]. The prone position has become a novel treatment for patients with COVID-19 who are awake and spontaneously breathing [167,168,169]. In awake, non-intubated patients, the prone position did not decrease lung ventilation inhomogeneity despite an improvement in oxygenation [170].

In addition, there was no difference in the regional ventilation distribution between the prone and supine positions during non-invasive ventilation. However, for patients under invasive ventilation, prone positioning led to a redistribution of ventilation to the dorsal regions, as demonstrated by the EIT. Oxygenation improved in the prone position with both invasive and non-invasive ventilation. They are likely to be governed by several underlying mechanisms [171]. Prone positioning with conventional oxygen therapy also increased oxygenation and improved global and regional end-expiratory lung impedance within less than 5 h [172]. Although awake proning appears to reduce the risk of tracheal intubation, it does not reduce mortality. However, clinicians are concerned that awake proning may worsen patient outcomes by increasing self-inflicted lung injury [173,174] or delaying tracheal intubation and invasive mechanical ventilation. However, there is a strong recommendation for a trial of awake proning in adult patients with COVID-19-related hypoxaemic respiratory failure who are not invasively ventilated [175]. It has also been described that in COVID-19 patients, the prone position decreased CL at low PEEP levels compared to the supine position. On the other hand, it increased the OD at a PEEP greater than 10 cmH_2_O [176]. Electrical impedance tomography was also used to evaluate the effects of different body positions on the regional lung mechanics and ventilation distribution. Sequential lateral positioning showed increased regional lung compliance and alveolar recruitment without increased airway pressure [177]. Furthermore, lateral positioning decreases overdistension in COVID-19-associated ARDS [178]. However, the ventilation distribution and response to lateral positioning vary among patients with spontaneous breathing. Individualised positioning should be customised using the EIT [179].

In spontaneously breathing patients, EIT is also used to evaluate or predict the effects of various therapies on lung mechanics in COVID-19 patients. For example, in a patient with a high-flow nasal cannula, the EIT was used to monitor changes in the EELI using different flows. The highest-end EELI resulted in the highest patient comfort level and the lowest respiratory rate [180]. Rauseo et al. used changes in EELI to predict the recruitment and failure of non-invasive continuous positive pressure ventilation (CPAP). Changes of less than 40% EELI during a single PEEP decrease in the supine position seemed to be a good predictor of poor recruitment and CPAP failure [181]. De Jongh et al. developed an algorithm using regional peak flow during a PEEP trial to titrate PEEP in COVID-19 patients on pressure support or CPAP [19]. This agrees well with the regional compliance-based approach for patients on controlled mechanical ventilation [17].

During the COVID-19 pandemic, EIT has also been used as a diagnostic tool, for example, for real-time visualisation of barotrauma risk [134], to visualise regional ventilation decrease during bronchoalveolar lavage and the recovery of regional ventilation afterwards [182], and to evaluate long-term dyspnoea by quantifying regional ventilation inhomogeneity in COVID-19 survivors during a one-year follow-up [183].

## 10. Conclusions

Electrical impedance tomography use has increased in clinical settings since the COVID-19 pandemic began. This has shifted the use of EIT from a research niche to a bedside clinical tool. At this time, bedside EIT was mainly used for PEEP titration. However, EIT has the potential to become the standard for respiratory monitoring of patients with or at risk of respiratory failure, with the aim of individualising therapy in various applications besides PEEP titration, such as optimal patient posture in spontaneously breathing patients or as a diagnostic tool. The major challenge for the future is to minimise the constraints and simplify decision making using algorithms.

## 11. Main Considerations

1. Use EIT as a non-invasive imaging modality to monitor lung function in critically ill patients.
2. EIT has shown promise in detecting changes in regional lung ventilation in response to interventions such as mechanical ventilation, prone positioning, and recruitment manoeuvres.
3. EIT can provide clinicians with real-time information regarding the distribution of ventilation in the lungs, which may help guide interventions and improve patient outcomes.
4. EIT has the potential to be used in clinical trials to evaluate the efficacy of interventions aimed at improving lung function in critically ill patients.
5. Further research is needed to establish EIT’s accuracy, reliability, and clinical utility of the EIT in critically ill patients.

## 12. Recommendations for Future Studies

First, further studies are required to determine the optimal EIT technique for different patient populations and clinical scenarios. This could involve comparing different EIT methods, such as continuous and intermittent monitoring or investigating the use of EIT in conjunction with other monitoring tools.

Second, future studies should focus on developing predictive algorithms based on EIT data that could help clinicians detect and prevent respiratory failure before it occurs.

Third, large-scale, multicentre studies are needed to evaluate the clinical effectiveness of EIT in critically ill patients. This could involve randomised controlled trials comparing EIT-guided ventilation strategies to the standard of care or investigating the impact of EIT on patient outcomes such as length of stay and mortality.

Fourth, further studies are needed to explore the potential long-term benefits of EIT in critically ill patients. For example, they investigated the impact of EIT-guided ventilation strategies on long-term respiratory function and quality of life.

Lastly, mortality is an accessible endpoint, but given that the ICU population is diverse, mortality is a challenging end goal. Considerations should be given to suitable outcome measures in ventilation studies.

## Figures and Tables

**Table 1:** Overview of key studies on the clinical use of electrical impedance tomography.

Aim	Method	Author, Year	Study Design	Sample Size	Population	Intervention	Main Findings	Limitations	Future Directions
Best PEEP	Compliance-based approach [17]	Hsu et al., 2021 [42]	Prospective randomised controlled trial	EIT: *n* = 42 PV curve: *n* = 45	Moderate-to-severe ARDS	EIT guided PEEP at the intercept point of cumulated alveolar overdistension and collapse. PV curve guided PEEP at the point of maximal hysteresis.	Lower driving pressure and higher surviving rate in the EIT group (69% versus 44.4%).	The selected PEEP was fixed for the first 48 h; more frequent PEEP titration might be valuable.	What is the effect of PEEP titration over a longer period of time
	Compliance-based approach	He et al., 2021 [40]	Prospective randomized controlled trial	EIT: *n* = 61 Lower PEEP/FiO_2_-table: *n* = 56	ARDS	EIT guided PEEP at the intercept point of cumulated alveolar overdistension and collapse. If the intercept was between two PEEP steps, the PEEP level with the lowest GI was selected. Control group: lower PEEP/FiO_2_-table	A non-significant decrease of 6% mortality and a significant improvement in organ function at day 2 in the EIT group.	The effect of prone positioning on the results and outcomes was not analysed.	Could early EIT-guided PEEP setting significantly decrease mortality in ARDS
	Compliance-based approach	Heines et al., 2019 [31]	Retrospective analysis	N = 39	ARDS	PEEP set at the intercept point of cumulated alveolar overdistension and collapse, compared to the ARDS network PEEP/FiO_2_-table and clinician-based PEEP.	In approximately two-thirds of the patients, EIT-guided PEEP differed from physicians’ set PEEP and from the ARDS network PEEP/FiO_2_-table.	Retrospective analysis without outcome data	Is EIT-guided PEEP superior to physicians’ set PEEP or the ARDS network table on oxygenation and respiratory mechanics
	EELI	Eronia et al., 2017 [49]	Feasibility study	N = 16	Acute hypoxic respiratory failure	PEEP was set after recruitment manoeuvres until EELI maintained stability over time.	EIT-guided PEEP was feasible and led to a higher PEEP level compared to the ARDS network PEEP/FiO_2_ table, resulting in improved oxygenation. and a decrease in driving pressure	EELI tracing could not successfully detect the PEEP level associated with sustained recruitment in 2 out of the 16 patients.	Does this strategy of titrating PEEP result in a decrease in ventilator-induced lung injury
	Regional peak-flow	de Jongh et al., 2023 [19]	An observational validation study in a prospective cohort	N = 78	COVID-19 ARDS	Cumulative overdistension and cumulative collapse rates are calculated based on the highest regional peak flow and validated with the compliance-based approach to use in spontaneously breathing patients.	The regional peak-flow approach showed good agreement with the compliance-based approach and, therefore, might be a valid method to quantify regional lung mechanics in spontaneously breathing patients.	Validation was only performed in COVID-19 patients.	Would ventilator settings based on this algorithm improve patients’ outcomes and reduce the weaning duration
Inhomogeneity	A/P ratio	Mauri et al., 2013 [67]	Prospective randomised cross-over study	N = 10	Mild-to-moderate ARDS	Evaluating ventral to dorsal changes in ventilation distribution while increasing/decreasing PEEP and/or PS.	Higher PEEP and lower PS increased the fraction of tidal ventilation reaching dependent lung regions.	The results may not apply to patients with severe ARDS.	What is the mechanism at the basis of the observed redistribution of ventilation (e.g., alveolar recruitment, increased diaphragm activity)
	CoV	Frerichs et al., 1998 [69]	Prospective observational study	N = 10	Patients scheduled for elective laparotomy	The CoV was calculated during spontaneous breathing and different modes of mechanical ventilation.	There are differences in ventilation distribution between spontaneous breathing and different ventilation modes. Ventilation distribution was larger in the dorsal lung during spontaneous breathing.	Only patients with healthy lungs were studied.	How does the CoV change in injured lungs and with different PEEP levels
	GI	Zhao et al., 2014 [86]	Retrospective analysis	ARDS: *n* = 18 Lung-healthy patients: *n* = 8	ARDS and lung-healthy	A constant low-flow inflation manoeuvre was performed. Recruited lung regions were identified where local impedance amplitudes exceeded 10% of the maximum amplitude during the manoeuvre.	GI highly correlates with lung recruitability.	The gold standard for identifying collapsed lung regions (CT) was missing.	Correlates GI also with alveolar recruitment in standard recruitment manoeuvres
	GI	Becher et al., 2015 [87]	Retrospective analysis	N = 9	ARDS	Lower and higher tidal volume was used at a PEEP level of 2 cmH_2_O below and 5 cmH_2_O above the lower inflexion point.	High tidal volumes may lead to a lower GI, especially at low PEEP settings.	Two different EIT devices were used, which may have influenced the results.	What is the influence of respiratory rate and posture on GI
Prevent atelectrauma	RVD	Wrigge et al., 2008 [18]	Randomised prospective experimental study	N = 16	Pigs with direct, indirect acute lung injury (*n* = 10) and healthy lungs (*n* = 6)	During slow inflation, simultaneous measurements of regional ventilation by EIT and dynamic CT.	RVD correlated well with recruited volume as measured by CT.	Experimental model, comparison with patients with lung injury should be made with caution. Control group was measured later in time and was, therefore, not randomised.	Develop a method to detect atelectrauma during normal (ongoing) mechanical ventilation
Oxygenation improvement using posture	Prone position	Spaeth et al., 2016 [104]	Prospective clinical study	N = 45	Patients with healthy lungs undergoing lumbar spine surgery	Patients were examined in the supine and prone position at a PEEP of 6, 9, and 12 cmH_2_O.	Commonly measured Crs do not reflect the differences in respiratory mechanics between supine and prone posture. Intra-tidal compliance profile revealed substantial differences in lung condition between both postures.	In prone position, chest and pelvis were supported with pads, which allows free movement of the abdomen and lower chest wall, causing lower intra-abdominal and intra-thoracic pressure. These findings only apply when free abdominal movements are ensured.	Is higher PEEP needed in prone position in affected lungs
	Prone position	Wang et al., 2022 [105]	Prospective physiological study	N = 10	ARDS	EIT evaluation at ignition of prone positioning, 3 h after and at the end of the first prone session	Increased ventilation in the dorsal regions without affecting ventral regions early after prone position. Resulting in increased PaO_2_/FiO_2_ ratio	The EIT assessment was only measured at 3 time points during prone position. Changes in ventilation distribution were not compared after turning to the supine position again.	What is the effect on ventilation distribution and oxygenation after prolonged prone positioning after turning back to the supine position
	Prone position and alveolar recruitment	Martinsson et al., 2021 [107]	Randomised controlled trial	N = 30	Patients after uncomplicated cardiac surgery	Alveolar recruitment manoeuvre in either the supine or the prone position.	Early after cardiac surgery, a lung recruitment manoeuvre in prone position improves oxygenation, dorsal ventilation and dorsal end-expiratory lung volume compared to the supine position.	There is a lack of FRC measurement after extubation.	What is the difference in effect on end-expiratory lung volume and oxygenation after a recruitment manoeuvre in patients with diseased lungs, comparing prone versus supine position
Diagnostic applications	Airway clearance	Garofalo et al., 2023 [122]	Physiological pilot study	N = 15	Tracheostomised patients undergoing mechanical ventilation	Use short HFPV cycles to investigate the effect on lung aeration and gas exchange.	Short cycles of HFPV superimposed on mechanical ventilation promoted alveolar recruitment and improved oxygenation in tracheostomised patients with high load of secretion.	Small sample size and a heterogeneous population. The definition of a hypersecretive patient is arguable.	Does HFPV increase oxygenation and ventilation in patients with atelectasis
	Tube placement	Steinmann et al., 2008 [125]	Feasibility study	N = 40	Patients requiring insertion of left-sided double-lumen tubes for one-lung ventilation during thoracic surgery	EIT was recorded during two-lung ventilation before induction of anaesthesia and after double-lumen tube placement and during one-lung ventilation in the supine and subsequently in the lateral position.	EIT enables online recognition of misplacement of left-sided double-lumen tubes in the contralateral main bronchus. However, as distribution of ventilation did not correlate with endobronchial cuff placement, EIT cannot replace fibreoptic bronchoscopy.	EIT was only used in the presence of left-sided double-lumen tubes.	Does EIT in right-sided double-lumen tube placement require additional definitions to account for the regional ventilation of the right upper lobe
	Pleural effusion	Rara et al., 2020 [131]	Prospective interventional study	N = 19 (6 excluded)	Ventilated patients with indication for pleural effusion drainage	Compare changes in EELI and EELV in response to the pleural effusion evacuation	The increase in EELI overestimated the increase in EELV, probably due to the removal of conductive effusion fluid	EELV is a global ventilation parameter, while EIT measurements are focused only on the cross-section within the wide plane of the belt	Estimating the amount of pleural fluid
	Pneumothorax	Yang et al., 2023 [135]	Retrospective cohort study	N = 203 (25 with PTX)	Mechanically ventilated patients who received EIT measurements in the supine position	Tidal impedance variation images were divided into four quadrants of equal size to track ventilation distribution in different regions of interest.	Regional ventilation defects can be observed in mechanically ventilated patients with PTX, requiring further diagnostics to confirm.	The baseline EIT in patients with a PTX before the onset was not recorded. Furthermore, the dynamic evolution of the PTX was not monitored.	Developing an algorithm that provides an alert in the presence of a PTX
	Pulmonary oedema	Zhao et al., 2019 [137]	Prospective observational study	N = 14	ARDS	Patients were rotated laterally along their longitudinal axis from the supine position to 45-degree left and right tilt to induce gravity-dependent redistribution of pulmonary oedema.	Postural changes did not reflect total extra-vascular lung water content.	Non-reproducible results may be introduced by measurement error of the trans-pulmonary thermodilution technique. No other reference technique was used.	Develop advanced measures to assess the level of pulmonary oedema
	Chronic lung diseases	Zhao et al., 2020 [140]	Prospective observational study	N = 25	Exacerbation of COPD and asthma	EIT measurements were conducted before and one hour after inhaling medication on two consecutive days.	Regional end-expiratory flow characterises air trapping, providing diagnostic information for monitoring the treatment of COPD and asthma patients.	No systematic clinical intervention was used to reduce air trapping.	What is the most effective way of medication nebulisation, and with which device

A/P ratio, anterior-to-posterior ventilation ratio; ARDS, acute respiratory distress syndrome; COPD, chronic obstructive pulmonary disease; CoV, centre of ventilation; Crs, respiratory system compliance; CT, computed tomography; EELI, end-expiratory lung impedance; EELV, end-expiratory lung volume; EIT, electrical impedance tomography; FiO_2_, fraction of inspired oxygen; FRC, functional residual capacity; GI, global inhomogeneity index; HFPV, high-frequency percussive ventilation; PaO_2_/FiO_2_ ratio, oxygen arterial partial pressure on inspired fraction of oxygen ratio; PEEP, positive end-expiratory pressure; PS, pressure support; PTX, pneumothorax; PV curve, pressure–volume curve; RVD, regional ventilation delay.

## Data Availability

Not applicable.

## Abbreviations

A/P ratio	anterior-to-posterior ventilation ratio
ARDS	acute respiratory distress syndrome
AU	arbitrary unit
Cdyn	dynamic respiratory system compliance
CL	alveolar collapse
CoV	centre of ventilation
CPAP	continuous positive airway pressure
CT	computed tomography
DLT	double-lumen tube
EELI	end-expiratory lung impedance
EIP	end-inspiratory pressure
EIT	electrical impedance tomography
GI	global inhomogeneity index
IR	impedance ratio
LL	lower limit
OD	alveolar overdistension
PEEP	positive end-expiratory pressure
P-SILI	patient self-inflicted lung injury
RPF	regional peak flow
RVD	regional ventilation delay
SARS-CoV-2	severe acute respiratory syndrome coronavirus 2
TID	tidal impedance difference
UL	upper limit
VALI	Ventilator-associated lung injury
